# Microalgae as bioreactors for bioplastic production

**DOI:** 10.1186/1475-2859-10-81

**Published:** 2011-10-17

**Authors:** Franziska Hempel, Andrew S Bozarth, Nicole Lindenkamp, Andreas Klingl, Stefan Zauner, Uwe Linne, Alexander Steinbüchel, Uwe G Maier

**Affiliations:** 1 LOEWE Research Centre for Synthetic Microbiology (SYNMIKRO), Hans-Meerwein-Strasse, 35032 Marburg, Germany; 2 Cell Biology, Department of Biology, Philipps-University of Marburg, Karl-von-Frisch-Strasse 8, 35032 Marburg, Germany; 3 Biochemistry, Department of Chemistry, Philipps-University of Marburg, Hans-Meerwein-Strasse, 35032 Marburg, Germany; 4 Institut für Molekulare Mikrobiologie und Biotechnologie, Westfälische Wilhelms-Universität Münster, Corrensstrasse 3, 48149 Münster, Germany

## Abstract

**Background:**

Poly-3-hydroxybutyrate (PHB) is a polyester with thermoplastic properties that is naturally occurring and produced by such bacteria as *Ralstonia eutropha *H16 and *Bacillus megaterium*. In contrast to currently utilized plastics and most synthetic polymers, PHB is biodegradable, and its production is not dependent on fossil resources making this bioplastic interesting for various industrial applications.

**Results:**

In this study, we report on introducing the bacterial PHB pathway of *R. eutropha *H16 into the diatom *Phaeodactylum tricornutum*, thereby demonstrating for the first time that PHB production is feasible in a microalgal system. Expression of the bacterial enzymes was sufficient to result in PHB levels of up to 10.6% of algal dry weight. The bioplastic accumulated in granule-like structures in the cytosol of the cells, as shown by light and electron microscopy.

**Conclusions:**

Our studies demonstrate the great potential of microalgae like the diatom *P. tricornutum *to serve as solar-powered expression factories and reveal great advantages compared to plant based production systems.

## Background

About 140 million tons of plastic are consumed every year worldwide, which necessitates the processing of approximately 150 million tons of fossil fuels and directly causes immense amounts of waste that can take thousands of years to naturally deteriorate, if it degrades at all [1]. Consequently, bioplastics are a feasible alternative in that they are not based on fossil resources and can easily be biodegraded. So far, however, production costs for petroleum-derived polymers still remain lower than biodegradable alternatives, which is a hindrance to commercial development and retail of environmentally friendly alternatives.

Poly-(R)-3-hydroxybutyrate (PHB) is an aliphatic polyester with thermoplastic properties, which is naturally produced by certain bacteria as storage compound and is 100% biodegradable [1-5]. PHB is synthesized from acetyl-CoA by the action of three enzymes: a ketothiolase, an acetoacetyl-CoA reductase and a PHB synthase [6]. Under optimal conditions bacteria such as *Ralstonia eutropha *H16 can produce up to 80% PHB of cellular dry weight, and some companies have specialized on commercial PHB production (e.g. Metabolix Inc., Micromidas Inc.). Nevertheless, costs for PHB production by bacterial fermentation are still very high, which brought plants into focus as photosynthesis fueled low-cost production system [7-10]. The three bacterial enzymes were expressed in the cytosol or targeted to different compartments of the plant cell leading to high amounts of PHB accumulation in the plastids of *Arabidopsis thaliana *(up to 40% of dry weight) [11,12]. However, due to stunted growth and infertility, these plants were not suitable for large-scale cultivation. Today, the highest levels of PHB synthesis in plants with fertile offspring are obtained in the plastid of *Nicotiana tabacum *resulting in up to 18% PHB of cellular dry weight [13].

In general, plant-based expression systems are very attractive in that no external organic carbon source is required, which is quite an important cost factor for large-scale production systems [14-16]. On the other hand, however, plant-based expression systems compete directly with subsistence crops for agricultural acreage and the dissemination of transgenic plants is difficult to control, which has been an ethical concern and led to strict regulatory controls of transgenic plants in many countries. Major drawbacks for the establishment of plant-based expression systems are the comparatively long growth rates resulting in a decrease in profitability. This casts a shadow on plant-based expression systems, rendering them unable to compete with the current well-established bacterial systems. Microalgae share all the advantages of photosynthetically driven eukaryotic systems but lack many of the mentioned disadvantages i.e. they possess high growth rates, are easy to handle and do not need much more than light and water for cultivation [17]. Thus, microalgae are thought to have great potential as novel low-cost expression systems especially if aiming at the biosynthesis of recombinant proteins needed in numerous industrial, therapeutic or diagnostic applications [18-21].

In this study, we present for the first time a report on the biosynthesis of a biotechnologically relevant biopolymer in a microalgal system. We demonstrate that production of the bioplastic PHB is feasible in the diatom *Phaeodactylum tricornutum *by introducing the bacterial PHB pathway into the cytosolic compartment. PHB levels of up to 10.6% of algal dry weight were obtained revealing the great potential of this low-cost and environmentally friendly expression system.

## Results and Discussion

**Figure 1:**
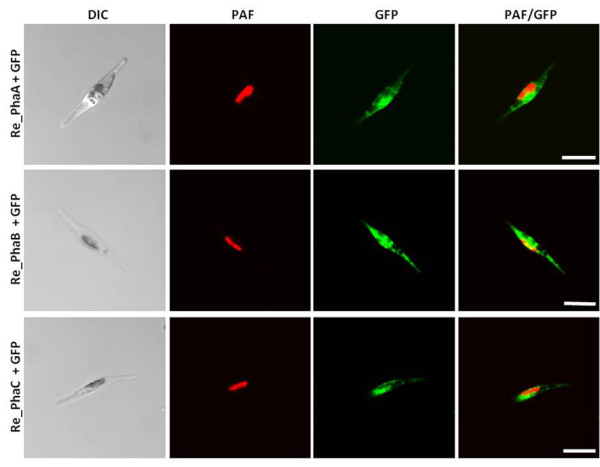
***In vivo *localization studies on PhaA, PhaB and PhaC expression in *P. tricornutum***. Sequences for PhaA (ketothiolase), PhaB (acetoacetyl-CoA reductase) and PhaC (PHB synthase) of *R. eutropha *H16 were introduced as GFP fusion proteins in *P. tricornutum*. All three enzymes were expressed in the heterologous system and accumulate in the cytosol as no specific targeting signal was added. Plastid autofluorescence is shown in red and GFP fluorescence is depicted in green. Scale bar represents 10 μm. PAF - plastid autofluorescence, Re - *R. eutropha *H16

**Figure 2:**
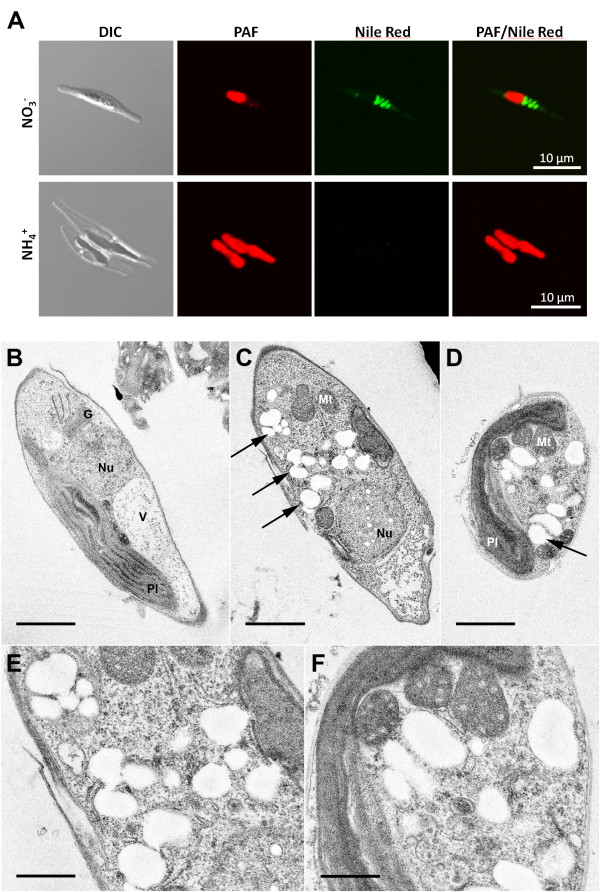
**Fluorescence and electron microscopic analyses on PHB accumulation in *P. tricornutum***. Cytosolic expression of enzymes PhaA, PhaB and PhaC of *R. eutropha *H16 induces the formation of granule-like structures that are stained by the lipophilic dye Nile red as visualized by fluorescence microscopy (A: NO_3_^-^). Under non-induced conditions no such granules were observed (A: NH_4_^+^). Electron microscopic analyses confirm cytosolic accumulation of electron-translucent granules (exemplarily marked by arrows) in cell lines expressing bacterial enzymes of the PHB pathway (C-F). PHB granules are about 0.1-0.3 μm in size and were not observed under non-induced conditions (B). Scale bar represents 1 μm (B-D) and 500 nm (E/F). DIC - differential interference contrast, G - golgi apparatus, Mt -mitochondrium, Nu - nucleus, PAF - plastid autofluorescence, Pl - plastid, V - vacuole

**Figure 3:**
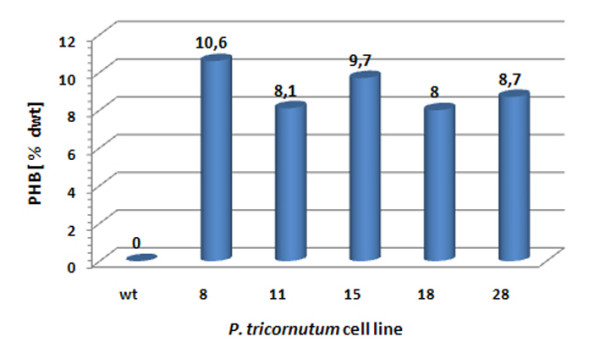
**Quantification of PHB synthesis in the cytosol of *P. tricornutum***. The level of PHB synthesis for five transgenic *P. tricornutum *cell lines (No. 8, 11, 15, 18, 28) was analyzed by gas chromatography coupled to mass spectrometry. After 7 days of PhaA/PhaB/PhaC expression PHB levels of 8.0 to 10.6% of algal dry weight were detected. Wild type cells were negative for PHB synthesis. dwt - dry weight, wt - wild type

The enzymes PhaA (ketothiolase), PhaB (acetoacetyl-CoA reductase) and PhaC (PHB synthase) of the Gram-negative bacterium *R. eutropha *H16 were expressed in the cytosol of the diatom *P. tricornutum *to test whether polyhydroxybutyrate (PHB) can be produced in a microalgal system. Initial *in vivo *localization studies with GFP fusion proteins demonstrated that all three enzymes are expressed in the heterologous system and accumulate within the cytosol (Figure 1). *P. tricornutum *cells that were co-transfected with sequences for all three enzymes being under the control of a nitrate-inducible promoter were first analyzed via PCR for the integration of all three constructs and were subsequently checked for morphological anomalies. Interestingly, when transferred to nitrate containing medium for 5-7 days (inducing the expression of PhaA, PhaB and PhaC) transfectants accumulated large amounts of granule-like structures within the cytosol, which were specifically labeled with the lipophilic dye Nile Red used in many other studies for *in vivo *staining of PHB granules (Figure 2A). Electron microscopic analyses confirmed these data demonstrating that cells were filled with granules that are not present in wild type cells or non-induced cells of the same line (Figure 2B-F). Gas chromatographic analyses of *P. tricornutum phaA*/*phaB*/*phaC*-transfectants transferred to nitrate-containing medium for 7 days revealed that such cells indeed accumulate PHB to levels of up to 10.6% of algal dry weight (Figure 3). Thereby, PHB accumulation turned out to be dependent on the induction period as only 1-3 days of PhaA/PhaB/PhaC expression resulted in much lower PHB quantities (data not shown). Importantly, wild type cells did not accumulate PHB by natural means (Figure 3).

The results of this study show for the very first time that PHB production is possible in a microalgae system. Interestingly, in comparison to efforts on PHB synthesis in the cytosol of plants, PHB expression levels in *P. tricornutum *are about 100-fold higher [7]. This might be due to large lipid deposits present in the cytosol of *P. tricornutum*, as these microalgae naturally produce valuable omega-3-fatty acids [22-24]. Therefore, the acetyl-CoA pool, which is the basis for PHB synthesis, might be notably high in the cytosol of *P. tricornutum *and hence enable very efficient PHB production. To circumvent acetyl-CoA limitations as a drawback for PHB production in plants, other cellular compartments were tested, and indeed plastids, which provide a high acetyl-CoA content because of fatty acid synthesis, turned out to produce much higher levels of PHB [11,12]. The best PHB synthesis levels in plants with fertile offspring thus far were achieved in tobacco with PHB contributing to 18% of dry weight [13]. Upon a first glance, this looks promising, however, taking production time as an important economic factor into account plants do poorly in direct comparison to *P. tricornutum*, which needs approximately two weeks to accumulate similar PHB levels reached by plants during a vegetation period of 3 months.

Of course PHB production in *P. tricornutum *cannot presently compete with bioplastic production in *R. eutropha*, which was established commercially many years ago. Nevertheless, this pilot experiment together with many other current projects on microalgal biotechnology highlights the immense potential of these photosynthetically driven production systems. Surely, such progress in microalgal biotechnology will boost the development of efficient photobioreactors for use in large-scale cultivation, which is currently one of the most limiting factors to put low-cost production into practice.

## Conclusions

Altogether, this study has demonstrated that microalgae like the diatom *P. tricornutum *have a great potential not only as biosynthetic factory for recombinant proteins but also as photosynthetically fueled bioreactors for synthesizing biotechnologically relevant polymers like PHB. Even though no enzyme engineering, no adaptations to *P. tricornutum *specific codon-usage, and no large-scale screening have been applied in these initial analyses, relatively high PHB levels of up to 10.6% of algal dry weight have been obtained. Thus, in the future, there will be a focus on various targets for enhancing PHB biosynthesis in *P. tricornutum*. Other subcellular compartments such as the plastids might yet be interesting sites for PHB synthesis. Diatoms are naturally rich in lipids and silicate and already have applications in biotechnology [17]. Hence, inserting and/or altering biochemical pathways in diatoms in order to synthesize complex molecules, biologically active substances, and raw materials may have a number of applications in for example the nanotechnology industry and the production of renewable biofuels.

## Methods

### Plasmid construction and *P. tricornutum *transfection

Plasmid pBHR68 [25] was used as template for amplification of *phaA*, *phaB *and *phaC *genes from *R. eutropha *H16. For *in vivo *localization studies sequences were cloned upstream to the eGFP (enhanced green fluorescent protein) sequence into the vector pPha-NR, which is a derivative of pPhaT1 with endogenous nitrate reductase promoter/terminator flanking the multiple cloning site [GenBank:JN180663]. The inducible nitrate reductase promoter system was established earlier in the diatom *C. fusiformis *by Poulsen et al. 2005 [26]. Transfection proceeded as described previously [27] with the exception that cells were grown under non-induced conditions with NH_4_^+ ^as sole nitrogen source. For PHB synthesis in *P. tricornutum*, the sequence for *phaC *was cloned into the vector pPha-NR (not containing eGFP), and sequences of *phaA *and *phaB *were inserted into the vector pPha-DUAL[2xNR], which is a pPha-NR derivative with two multiple cloning sites both under the control of endogenous nitrate reductase promoter [GenBank:JN180664]. Both plasmids were mixed and co-transfected under non-induced conditions.

### Cell culture and induction of recombinant protein expression

Cells were grown in f/2 medium under standard conditions as described elsewhere (Apt et al. 1999) with either 0.9 mM NO_3_^- ^or 1.5 mM NH_4_^+ ^as the nitrogen source. For *in vivo *localization studies on GFP fusion proteins transfectants were grown in media containing NO_3_^- ^to induce recombinant protein expression. After 3 days, clones were analyzed by confocal laser scanning microscopy. *PhaA*/*phaB*/*phaC *co-transfectants were first determined to have genomic integration by colony PCR for all three sequences. Subsequently, positive colonies were grown in liquid culture containing NH_4_^+ ^and allowed to reach exponential phase, whereupon they were transferred to NO_3_^- ^containing medium for varying time periods. For visualization of PHB granules, cells were induced for 5 days and analyzed by electron and confocal microscopic analyses, respectively. For confocal microscopy, cells were pre-incubated with the lipophilic dye Nile Red (0.5 μg/ml) for 24 h.

### PHB analyses

For analyses on PHB production cultures were grown in NH_4_^+ ^containing medium, washed in nitrogen-free medium and transferred to NO_3_^- ^containing medium for 7 days. Cells were harvested (1500 × *g*, 10 min), washed with phosphate buffered saline (PBS) and lyophilized for 24 hours. The PHB contents of the cells were determined upon methanolysis of 5 to 10 mg lyophilized cells in presence of 2 ml methanol/sulfuric acid (85:15, v/v) and 2 ml chloroform. The resulting methyl esters of 3-hydroxybutyrate were analysed by gas chromatography using an Agilent 6850 GC (Agilent Technologies, Waldbronn, Germany) as described previously [28,29].

### Fluorescence and electron microscopy

*In vivo *localization of GFP fusion proteins was analysed with a confocal laser scanning microscope Leica TCS SP2 using a HCX PLAPO 63x/1.32-0.6 oil Ph3 CS objective. GFP, chlorophyll and Nile Red were excited at 488 nm, and fluorescence was detected at a bandwidth of 500-520 nm, 680-720 nm and 580-600 nm, respectively. For electron microscopic analyses, cells were centrifuged at 2000 × g for 5 min followed by cryo-fixation and resin embedding. The samples were high-pressure frozen in a Leica EM-PACT 2 and subsequently freeze substituted (Leica EM AFS 2; Leica, Vienna, Austria) with pure acetone containing 2% (w/v) osmium tetroxide, 0.1% (w/v) uranyl acetate and 5% (v/v) H_2_O. Freeze substitution was carried out at -90°C for 4 h, -60°C for 8 h, -30°C for 8 h and held at 0°C for 3 h with a heating time of 1 h in between each step. After washing the samples with ice cold acetone for three times and infiltration in Epon 812 (Ted Pella, Inc., USA) for 24 h, the resin was polymerized at 60°C for 72 h. Ultrathin sections were cut with a Leica Ultracut (Leica, Vienna, Austria), mounted on uncoated 400 mesh copper grids and post-stained with 2% (w/v) uranyl acetate for 20 min and 0.5% (w/v) lead citrate for 1 min. Transmission electron microscopy was carried out on a JEOL 2100 TEM operated at 80 kV in combination with a fast-scan 2 k × 2 k CCD camera F214 (TVIPS, Gauting, Germany).

## Competing interests

The authors declare that they have no competing interests.

## Authors' contributions

Research was designed by UGM, ASB and FH. NL conducted experimental work on PHB-analyses and AK performed electron microscopic analyses. FH is responsible for design and drafting of the manuscript and carried out further experimental work. AS, UL and SZ assisted in data analysis and review of the manuscript. All authors read and approved the final manuscript.
